# Supplementary material for: Transcriptional profiling of macrophages derived from monocytes and iPS cells identifies a conserved response to LPS and novel alternative transcription
Source: Sci Rep. 2015 Jul 30;5:12524. doi: 10.1038/srep12524 (PMC4519778; doi:10.1038/srep12524)
Supplement: Supplementary Information [file srep12524-s1.pdf]

Supplementary Information

Transcriptional profiling of macrophages derived from monocytes and iPS cells identifies a conserved response to LPS and novel alternative transcription

Kaur Alasoo, Fernando O. Martinez, Christine Hale, Siamon Gordon, Fiona Powrie, Gordon Dougan, Subhankar Mukhopadhyay, Daniel J. Gaffney

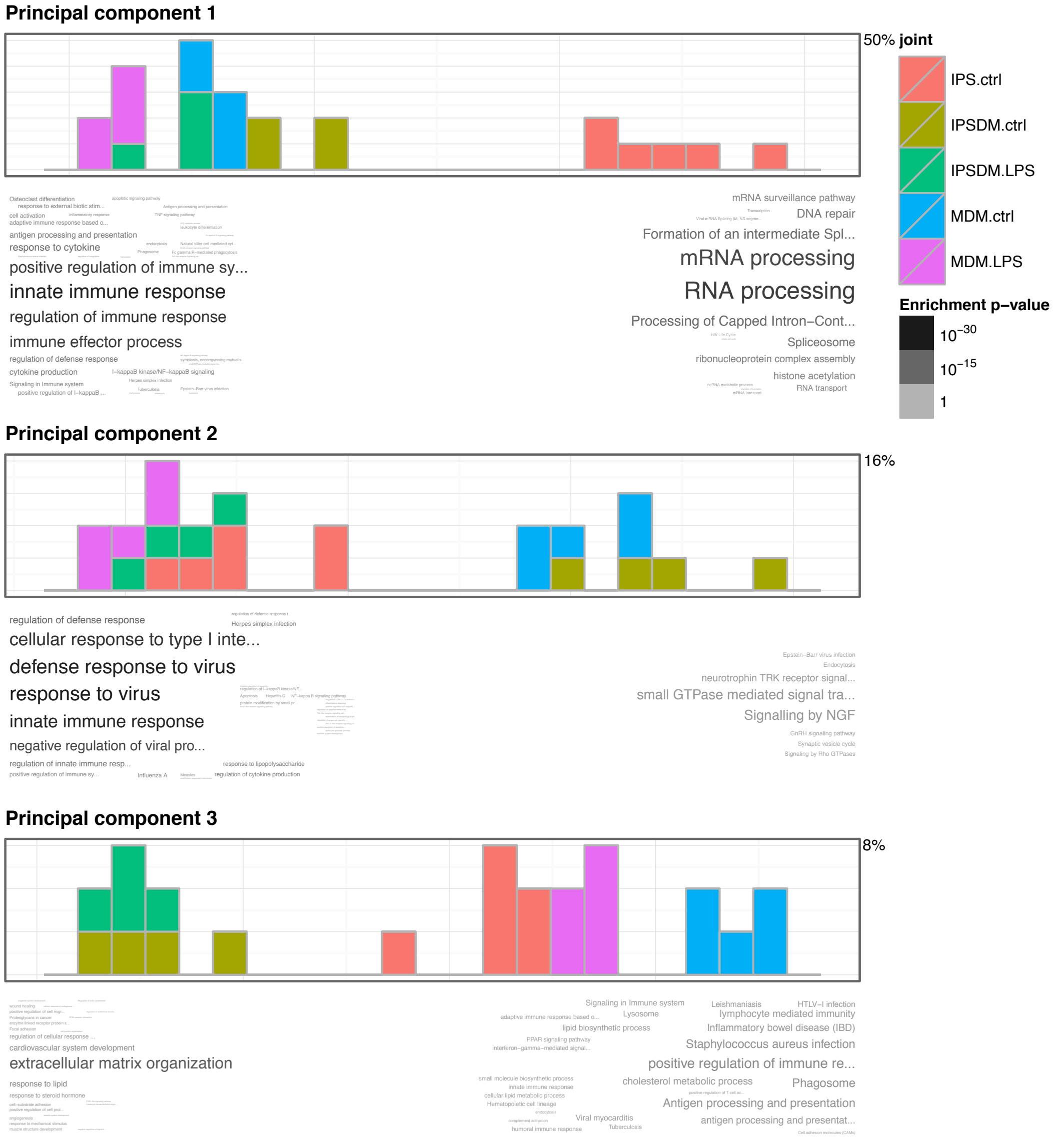

Supplementary Figure S1: Gene Ontology terms enriched in the first three principal components. PCA analysis on gene expression levels was performed as described in the main text. The results were then fed into GOsummaries R package to identify Gene Ontology categories that were enriched among genes with high loadings in each principal component.

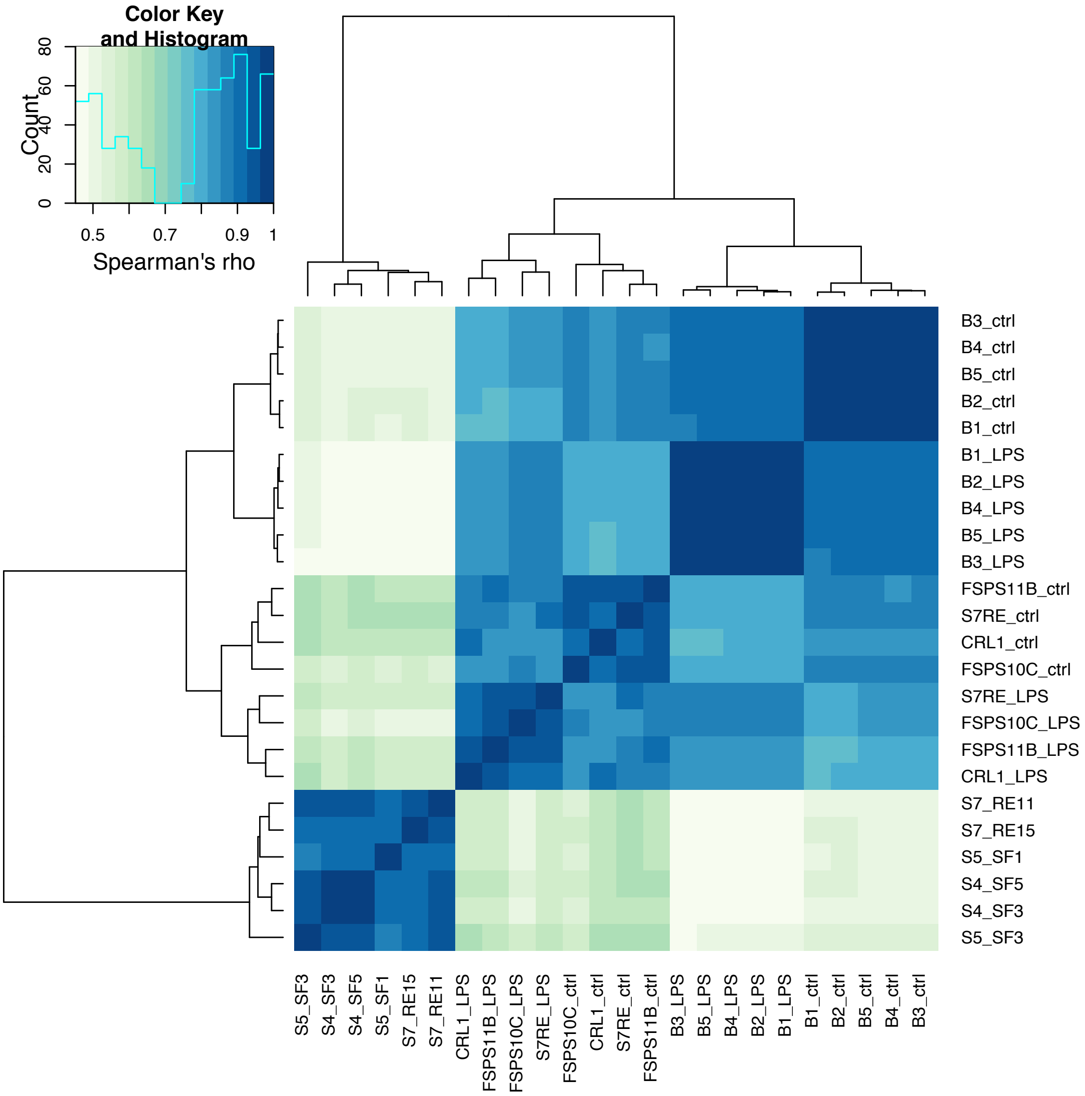

Supplementary Figure S2: Heatmap of pairwise Spearman's rank correlation between expressed genes (mean TPM > 2) in all RNA-Seq samples together with hierarchical clustering. B1-B5 represent five blood donors and FSPS11B, S7RE, FSPS10C and FSPS11B are the four iPS lines used for IPSDM differentiation. Control or LPS-treated samples are marked by _ctrl and _LPS suffixes, respectively. The remaining six samples (S7_RE11, S7_RE15, S5_SF1, S5_SF3, S4_SF5, S4_SF3) mark the undifferentiated iPS cells. IPSDMs are much more similar to MDMs than they are to iPS cells. Naïve and LPS-stimulated samples cluster separately within each macrophage type.

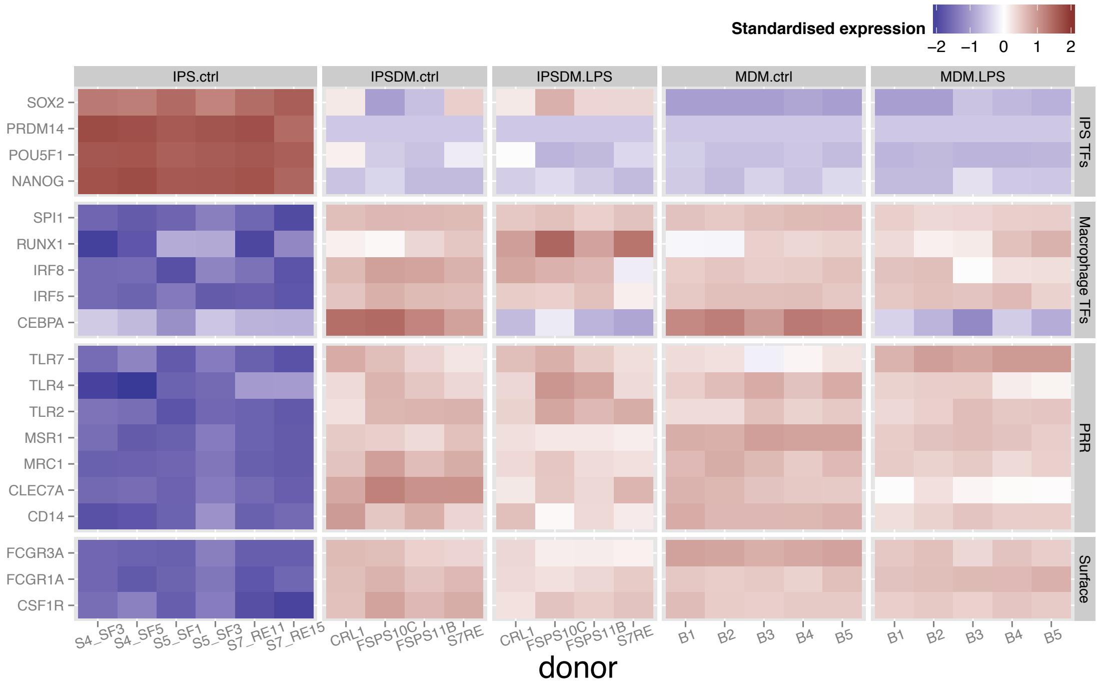

Supplementary Figure S3: Heatmap showing the gene expression of selected iPS-specific transcription factors (TFs), macrophage specific TFs, pattern recognition receptors (PRRs) and canonical macrophage cell surface markers. Rectangles correspond to measurements from independent biological replicates.

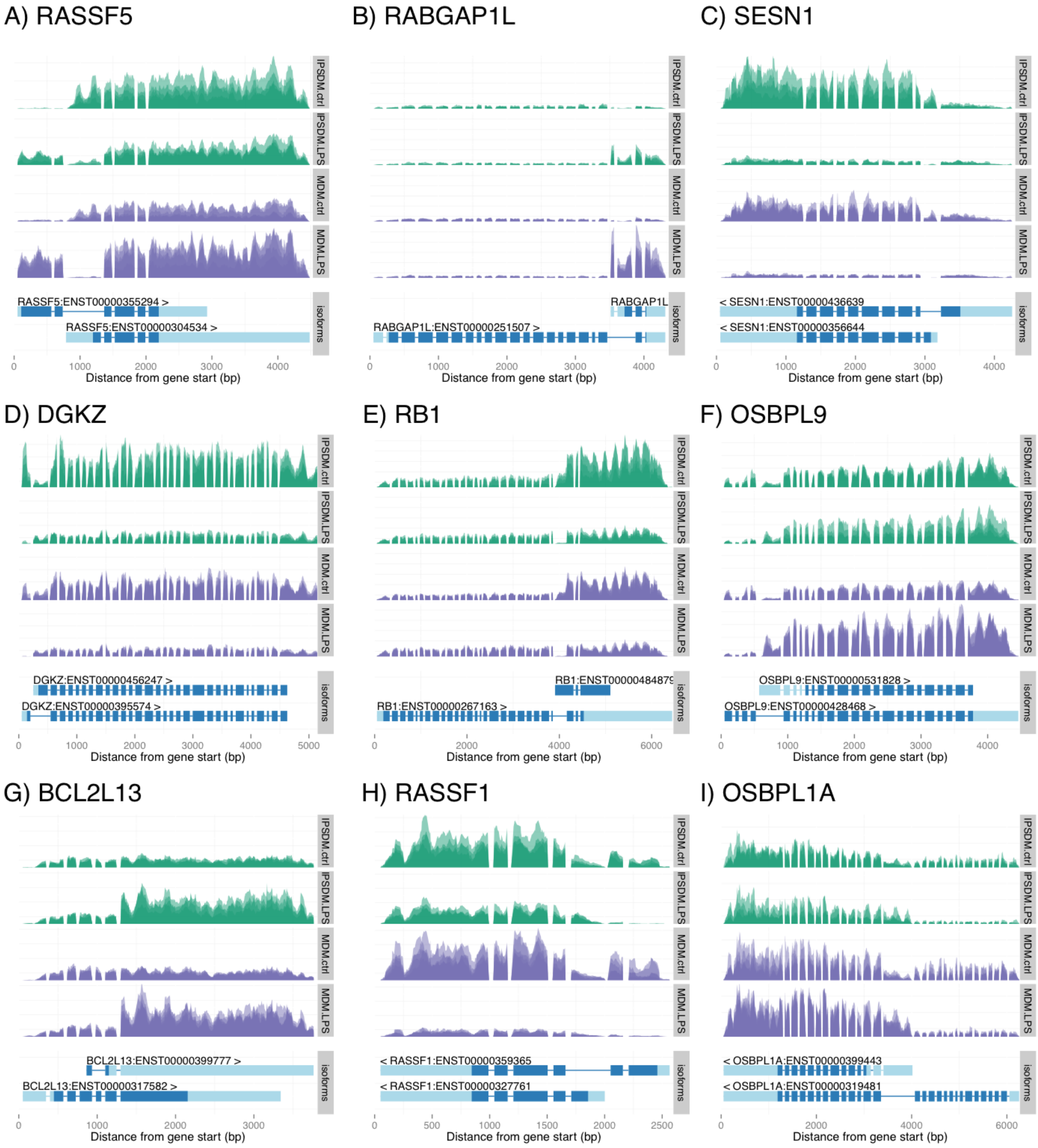

Supplementary Figure S4: Example genes showing strong alternative promoter usage in LPS response. Each panel shows normalised read depth across the gene body and flanking regions in IPSDMs (green) and MDMs (purple) with gene structure in the panel beneath each plot. Introns have been compressed relative to exons to facilitate visualisation.

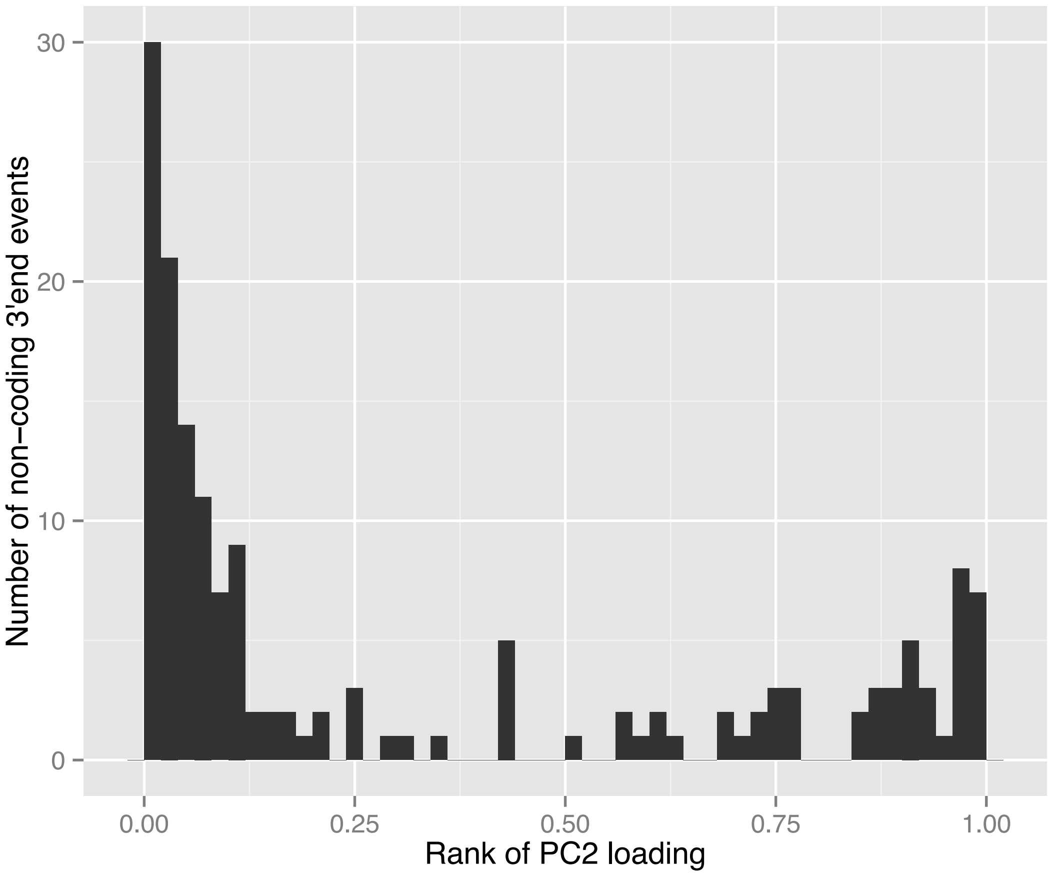

Supplementary Figure S5: Distribution of principal component 2 (PC2) weights for genes with 3' UTR changes either in LPS response or between MDMs and IPSDMs (162 genes). PCA on transcript proportions was performed as described in the main text. All genes were then ranked based on their weights in PC2 (from positive to negative). In PCA, genes with very large positive or negative weights within a given PC contribute strongly to the observed separation of samples in that PC. The ranks of the 3' UTR genes are significantly skewed towards 0 and 1 compared to a random sample of genes that should be uniformly distributed (chi-square goodness-of-fit test on binned ranks $X^2(9, N=162) = 330.10$, $p < 2.2 \times 10^{-16}$), indicating that they contribute strongly to PC2.

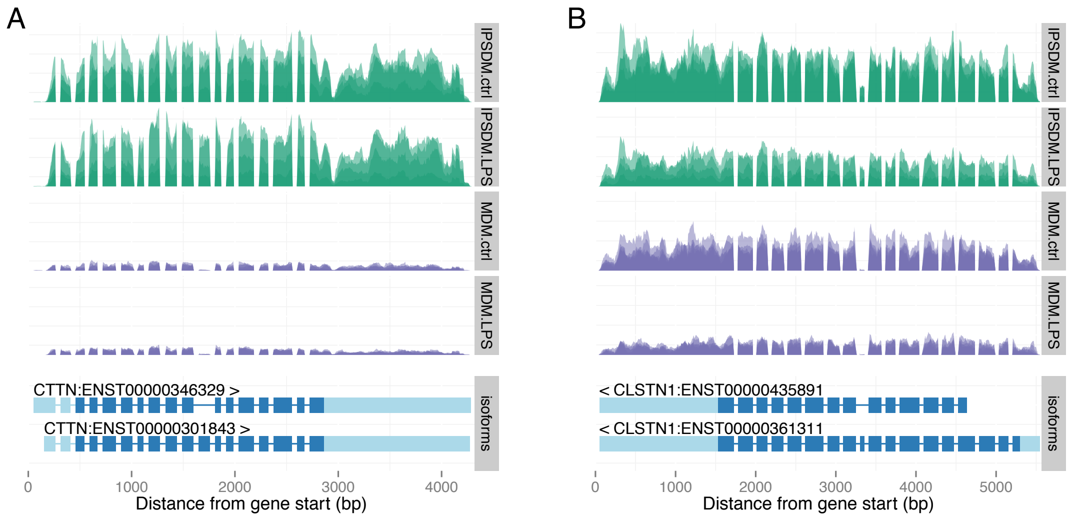

Supplementary Figure S6: Examples of alternative splicing between MDMs and IPSDMs. (a) Read coverage plot of the CTTN gene. Middle exon is differentially included between MDMs and IPSDMs. (b) Read coverage plot of the CLSTN1 gene. Middle exon is differentially included between MDMs and IPSDMs. Each panel shows normalised read depth across the gene body and flanking regions in IPSDMs (green) and MDMs (purple) with gene structure in the panel beneath each plot. Introns have been compressed relative to exons to facilitate visualisation.

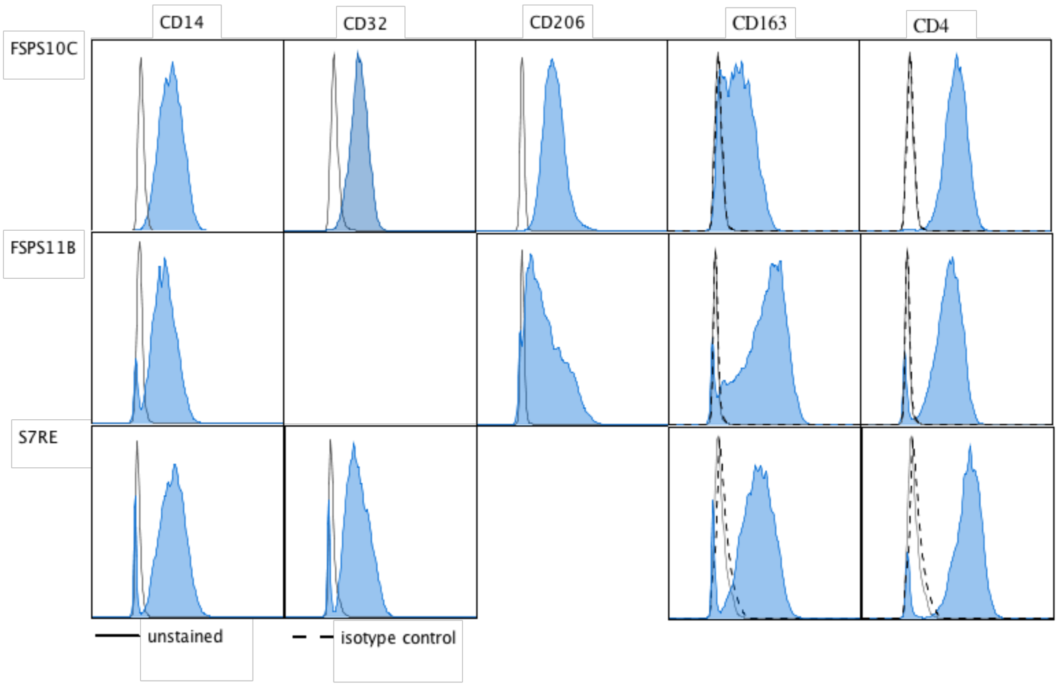

Supplementary Figure S7: Expression of five cell surface markers in macrophages derived from three independent IPS lines measured using flow cytometry. Blue indicates the population of cells stained for each surface marker. Solid and dotted lines correspond the unstained or isotype controls, respectively.

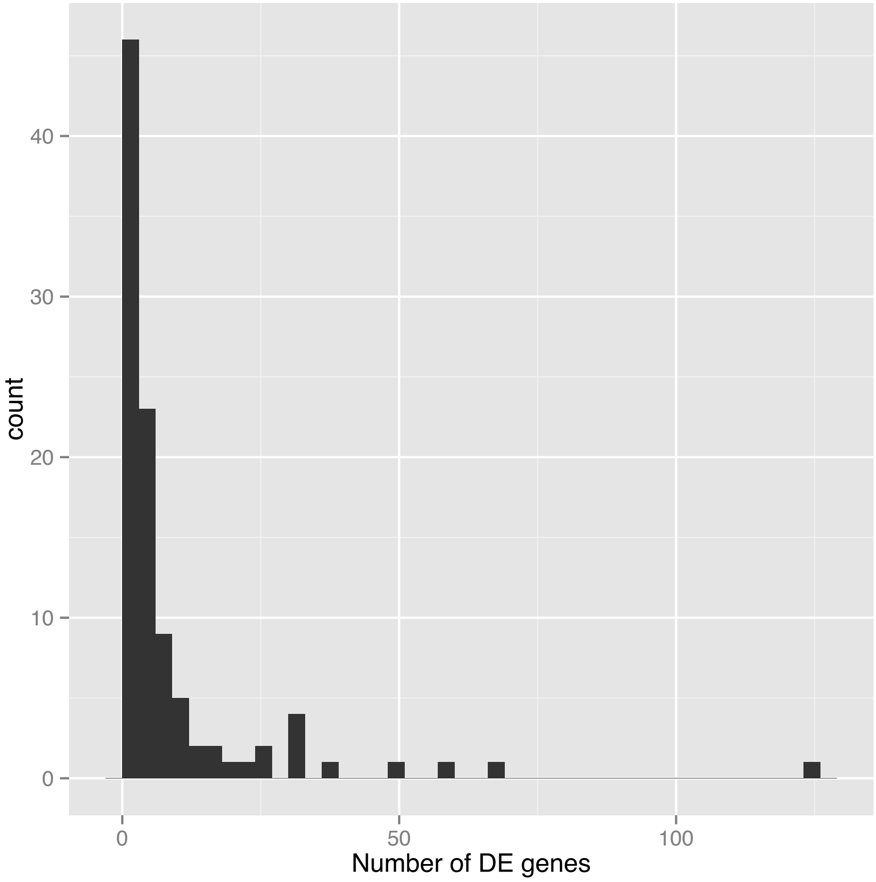

Supplementary Figure S8: Histogram of the number of differentially expressed genes between two groups of randomly selected individuals. We reanalysed published RNA-Seq data from from 84 British individuals (Lappalainen *et al*, Nature 2013). To mimic our experimental design, we repeatedly (100 times) sampled 9 individuals from the pool of 84, assigned them into two groups at random (four and five individuals) and used DEseq2 to estimate the number of differentially expressed genes between the groups that satisfied the same thresholds that we used in our main analysis (FDR < 0.01, fold change > 2). The median number of differentially expressed genes between the two groups was 3 and in 95% of the cases the number was less than 38.

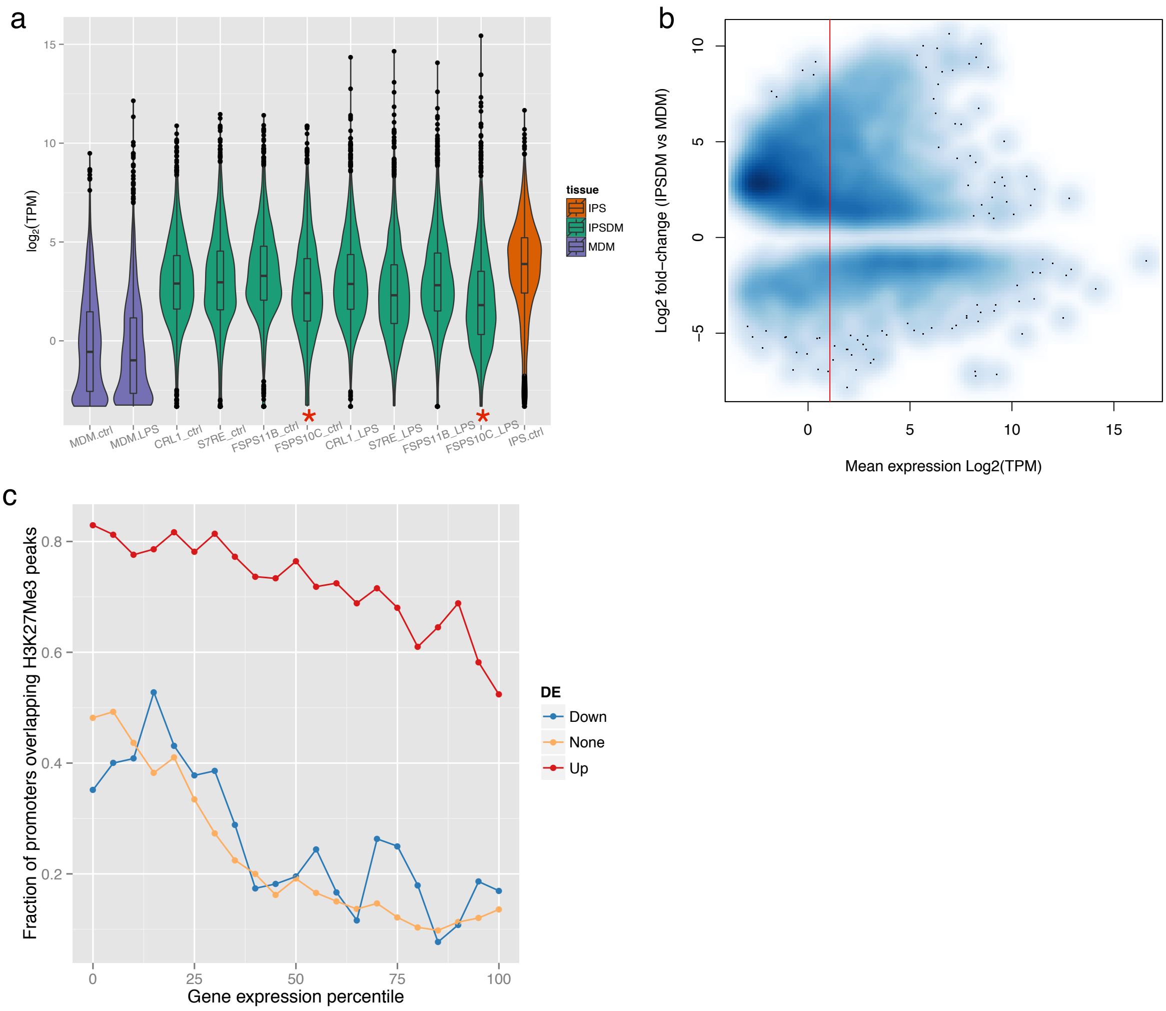

Supplementary Figure S9: (a) Expression levels of genes more highly expressed in IPSDMs compared to MDMs (TPM > 2). Purple violin plots show the mean expression of these genes in MDMs and orange in IPS cells. Red asterisks mark IPSDM samples (FSPS10C) that stained > 99% positive for CD14, CD206 and CD4 while S7RE and FSPS11B samples were ~91% positive.

(b) MA-plot of differentially expressed genes between MDMs and IPSDMs (without TPM cut-off). On the y-axis is the DESeq2 estimate of fold-change between MDMs and IPSDM. Red line denotes the 2 TPM cut-off used in most analyses. Most of the genes more highly expressed in MDMs have mean expression > 2 TPM. On the other hand, large proportion of genes more highly expressed in IPSDMs has extremely low mean expression level.

(c) Fraction of gene promoters overlapping H3K27Me3 peaks in ENCODE CD14+ monocyte samples stratified by the percentile of gene expression level. Genes upregulated in IPSDMs (red) were strongly enriched for repressive H3K27Me3 chromatin marks at their promoters compared to genes that were either downregulated (blue) or not differentially expressed (orange).

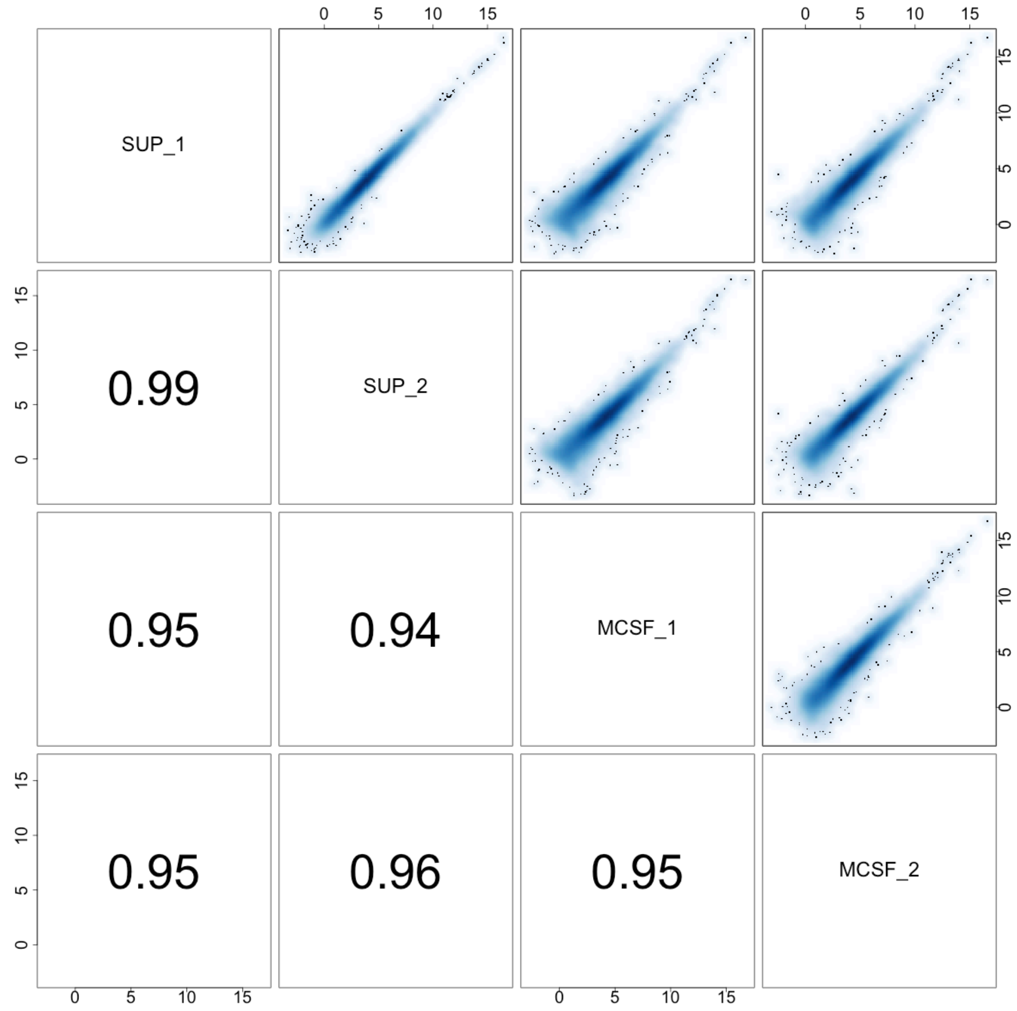

Supplementary Figure S10: Comparison of gene expression between two biological replicates of FSPS10C-derived IPSDMs differentiated with either supernatant (SUP_1 and SUP_2) or recombinant M-CSF (MCSF_1 and MCSF_2). Above diagonal: Pairwise scatterplots of expressed genes (TPM > 1) between all four samples. Below diagonal: Pairwise Spearman's correlation of gene expression between all four samples. The variation between supernatant and recombinant M-CSF samples is comparable to the variation between two biological replicates of recombinant M-CSF samples**.**

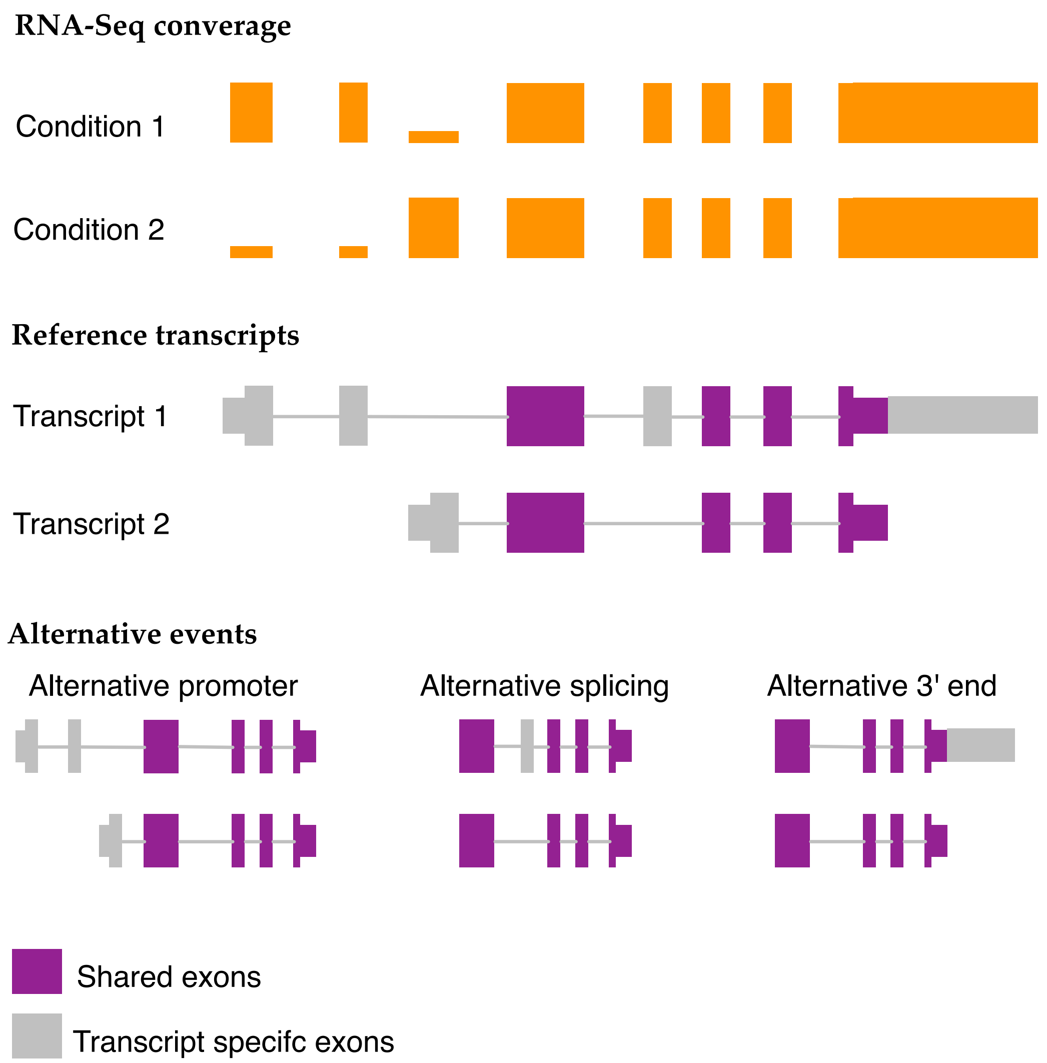

Supplementary Figure S11: Constructing alternative transcription events from alternative transcripts. Top panel shows RNA-Seq coverage over a gene suggesting alternative promoter usage between conditions 1 and 2. Middle panel shows hypothetical transcripts detected to be differentially expressed in the same conditions. Due to inaccurate reference transcript annotations the second promoter is always linked to skipped exon 4 and short 3' UTR. Comparing these two transcripts to each other gives a wrong impression that exon 4 and 3' UTR are also differentially expressed. Lower panel shows alternative transcription events constructed from the two transcripts using reviseAnnotations. Estimating the expression of these alternative events separately correctly identifies that only the promoter usage changes between conditions.
